# Effectiveness of Immersive Videos in Inducing Awe: An Experimental Study

**DOI:** 10.1038/s41598-017-01242-0

**Published:** 2017-04-27

**Authors:** Alice Chirico, Pietro Cipresso, David B. Yaden, Federica Biassoni, Giuseppe Riva, Andrea Gaggioli

**Affiliations:** 1 Università Cattolica del Sacro Cuore, Department of Psychology, Largo Gemelli, 1, 20123 Milan, Italy; 2 IRCCS Istituto Auxologico Italiano, Applied Technology for Neuro-Psychology Lab, via Magnasco, 2, 20149 Milan, Italy; 3 University of Pennsylvania, Department of Psychology, 3701 Market Street Suite 200, Philadelphia, PA 19104 USA

## Abstract

Awe, a complex emotion composed by the appraisal components of vastness and need for accommodation, is a profound and often meaningful experience. Despite its importance, psychologists have only recently begun empirical study of awe. At the experimental level, a main issue concerns how to elicit high intensity awe experiences in the lab. To address this issue, Virtual Reality (VR) has been proposed as a potential solution. Here, we considered the highest realistic form of VR: immersive videos. 42 participants watched at immersive and normal 2D videos displaying an awe or a neutral content. After the experience, they rated their level of awe and sense of presence. Participants’ psychophysiological responses (BVP, SC, sEMG) were recorded during the whole video exposure. We hypothesized that the immersive video condition would increase the intensity of awe experienced compared to 2D screen videos. Results indicated that immersive videos significantly enhanced the self-reported intensity of awe as well as the sense of presence. Immersive videos displaying an awe content also led to higher parasympathetic activation. These findings indicate the advantages of using VR in the experimental study of awe, with methodological implications for the study of other emotions.

## Introduction

The sight of stars, the image of earth from orbit, or witnessing childbirth are all experiences often elicit the emotion of awe. Awe is associated with deep feelings of wonder, astonishment, and sometimes fear – it can be both pleasurable and uncomfortable or overwhelming^1–3^. Awe is often involved when individuals face something that forces them to adjust their mental schema and to look for new patterns^1, 4^. According to Keltner and Haidt^1^ awe is induced by stimuli that are both vast and difficult to accommodate. Awe stimuli include vast and grand panoramas and sweeping vistas, as well as “grand theories” or big ideas.

Psychological study of awe has begun relatively recently. One of the main challenges concerning the experimental study of awe is the question of how to induce an intense feeling of this emotion in controlled settings^5, 6^. Previous research has suggested that the intensity of the induced emotion changes according to the specific induction procedure^7^. Recently, virtual reality (VR) has been proposed as a new technique to induce awe^5^. VR offers several theoretical advantages for eliciting and investigating high-intensity awe experiences. First, previous research has shown that VR-based perceptual stimulations – such as visual, auditorial, tactile and, when feasible, olfactive - can trigger strong emotional reactions^8–10^. Furthermore, VR is known to convey a sense of presence - the feeling of “being there” within an environment^11, 12^ – which has been found positively associated with the intensity of emotions experienced by participants^8, 9, 13^. From a methodological viewpoint, VR offers high degree of ecological validity: it allows researchers to simulate real-life contexts and situations, which can be used to study participants’ behaviours in highly-controlled laboratory conditions.

Here we tested the potential of immersive videos - the highest realistic form of Virtual Reality that excludes the dimensions of navigation^14^ - in inducing awe. We compared psychological and physiological responses to immersive displays of both awe inducing and neutral stimuli with the same content displayed on normal 2D screens. We hypothesized that immersive videos would be more effective than 2D screens at inducing awe, as measured through self-report and physiological measures.

## Awe

Awe includes at least three subcomponents that differentiate it from other emotions. First, awe is induced from stimuli characterized by vastness and a need for accommodation – stimuli features unique to awe^1, 4, 15^. Second, awe is composed of several sub-components such as wonder, joy, fear, and reverence^1, 15^. Consequently, awe can encompass a positive and/or a negative valence: positive awe^4^ and/or negative awe^15^. Third, awe can diminish the sense of self, that is, participants report a sense of smallness in the face of something perceived as and larger than one’s self^15, 16^. This change to one’s sense of self mediates awe’s ability to increase prosocial behaviours towards strangers^15, 17^.

Awe affects a number of cognitive processes. For example, awe broadens attention^18^, increasing awareness of others^17^. Awe can also alter time perception^19^ and can lead to a decreased estimation of one’s body size^20^. Further, experimentally-induced awe leads to greater feelings of uncertainty^21^. Finally, awe increases feelings of connectedness with other people and can lead to greater satisfaction towards life^3^. In particular, the disposition to live awe frequently prevents from several health risks such as depression or stress-related disorders^22^.

## Inducing awe in the lab

Inducing sufficiently intense awe experiences in the lab is a key challenge for researchers^5, 6^. Several emotion induction procedures have been used in previous research. Personal narratives consist of asking participants to recount first-hand experiences of awe vocally or in writing. This methodology has been effective for exploring several nuances awe, e.g., refs 4, 15, 23. A similar technique consists in asking participants to read short stories about prototypical awe elicitors, such as a sunset or a beautiful panorama^15^. Other techniques include the use of awe-induction images, such as grand views or natural phenomena, e.g., refs 6, 24. A more effective version of this technique relies on the use of videos, e.g., refs 15, 17, 25. Finally, awe has also been induced in experimental settings by exposing participants to natural environments (for example, a groove of eucalyptus; ref. 15 or buildings, e.g., refs 16, 26.

While these techniques have been generally effective in inducing awe, they have important limitations. Personal narratives generate recalled emotions that can be drastically different from those actually felt during the original event^27–29^. This limitation is even more crucial in recalling awe experience, since awe is a complex emotional state characterized by a mix of sub-components. Moreover, awe can comprise both positive and negative feelings^1, 4^. Image and video-based awe induction techniques have the advantage of using standardized stimuli and offer high degree of control on the experimental setting. On the other hand, these techniques typically generate low-intensity emotions that may have limited ecological validity^6^. This limitation can be overcome by exposing participants to real-life awe-induction scenarios. However, this technique may be unpractical and requires careful control of potential intervenient factors.

In summary, several experimental procedures can induce awe. However, the challenge remains of how to increase the intensity of awe in controlled conditions in order to reproduce the complex processes that characterize the emotion. As Ellard, Farchione, and Barlow^30^ suggested, emotion induction techniques should be able to set “complex associated processes in motion” (p. 233) not only inducing that emotion, but also the “*quality* of the associated experience” is crucial (p. 233, italics in the original). In particular, in the case of awe, the authors stress the importance of inducing the genuine and high-intensity emotion outlined by qualitative reports and depicted in literature or art^5, 6^.

Assessing awe in experimental settings is another methodological challenge. Researchers have generally used psychometric self-report measures of awe. Problematically, however, many studies use single item measures of awe. There are exceptions, such as Schurtz *et al*.^23^ who assessed awe through a more complex self-reported measure that addressed the “vastness” and the “need for accommodation” component. However, a validated retrospective measure of awe addressing all its subcomponents does not yet exist. On the other hand, some researchers have used physiological measures. Oveis *et al*.^31^ and Shiota *et al*.^24^ used the valence-arousal model of emotions^32, 33^ which differentiates affective states according to two dimensions of physiological arousal and hedonic valence. They found evidence for a sympathetic withdrawal during awe. In general, combining both conventional retrospective self-reports with physiological assessment would be better than either on its own.

## Virtual reality as awe-induction technique: the role of presence

VR is a technology that creates the perception of entering computer-generated interactive environments. Often, users can navigate these virtual spaces as if they were real physical spaces. This is achieved by combining different types of displays and stimuli (i.e. visual, auditory, tactile/haptic and sometimes gustative and olfactive) with sensors (i.e. head-tracking, hand tracking etc.) and controllers (hand held, treadmills for walking, etc.). Recently, VR has become of great interest for psychological researchers because of its ability to simulate real-life experiences in a controlled and safe laboratory setting. A further advantage of VR as an experimental tool is its ability to track participants’ behavior while exercising a high level of control on the stimuli delivered to the user. Finally, many forms of VR allow navigating inside the virtual environment by interacting with it. Because of these features, researchers can manipulate several parameters and variables of the participants’ simulated environment, according to the specific needs of the experimental design.

VR has already been used in some emotion-induction studies, for both clinical applications and basic research. In clinical applications, VR has been effective in inducing negative emotional states, such as fear and anxiety. For example, in VR-based treatment of phobias, simulated versions of threatening stimuli have been used to elicit the phobic responses and gradually extinguish them^9^. VR has also been used in stress-inoculation protocols to simulate and manage stressful situations^9, 10, 34^. Finally, VR has been used to explore affective and perceptual layers of specific pathologies, e.g., refs 35, 36. In non-clinical settings, VR has been successfully used as mood induction procedure (MIP) to elicit various discrete emotions^8, 37–39^. For example, Felnhofer *et al*.^38^ were able to elicit specific affective states (joy, sadness, boredom, anger and anxiety) by exposing participants to different virtual park scenarios which differed in terms of weather, facial expressions on other people, music, and other parameters. The sense of presence, or the illusion of “being there” created by a virtual (or physical) world, is an important element in eliciting strong emotional responses^11, 12^. Although different theoretical models of presence have been proposed (for a review, see refs 40, 41), most authors agree that presence is a multidimensional phenomenon encompassing several sub-components^40, 42^. The first component is the *feeling of physical space*, i.e. the perception of being transported in another physical space. The second component is perceptual realism, which is how much the virtual stimuli resemble the real ones on which they are modelled^10, 40^. A further component is the extent to which users can feel surrounded by the environment, or the feeling of *immersion* ^9, 40^. This dimension is brought about by the capacity to experience a virtual environment from a immersive perspective and sensorial isolation from the real world^9^. Finally, a crucial component of presence is the degree of interest in a given virtual environment, i.e. their level of *engagement* ^43^.

These components of presence are generally associated to the intensity of emotions reported by participants. For example, Baños and colleagues^8^ found that more emotional content displayed in a virtual immersive environment increases *engagement* ^43^. Baños *et al*.^13^ found that both *engagement* and *physical space* components positively correlated with emotional intensity. Further, the component of *immersion* within a virtual setting increases the intensity of the emotional reaction^9, 40^. Generally, it emerged that the more immersive the VR experience is, the higher the levels of presence reported by participants^40^.

## The current study

Previous attempts to elicit awe using VR environments have led to promising qualitative results^44, 45^ and while the capacity for VR to enhance quantitative measures of awe has been theorized^5^, it had not yet been directly tested experimentally. In the current study, we tested the potential of one of the highest realistic form of VR that excludes the dimensions of navigation with the virtual environment^14^. We induced strong feelings of awe by immersing participants in immersive video displays of vast and panoramic scenes of natural beauty from a 360° perspective.

The immersive video is a new video format typically recorded through an apparatus of multiple cameras, or using a specific VR camera composed of several camera lenses embedded into the device. It is used a specific software to integrate the raw footage into a coherent surrounding scene displayed on a Head Mounted Display^14^. This format is characterized by high degree of pictorial realism and the capacity to move around exploring the environment from an immersive perspective provides a strong illusion of depth, which allows for more immersive experiences. All these elements can support a strong feeling of presence, which in turn, can contribute to elicit a more intense emotion of awe. The main difference between immersive videos and the so-called 2D videos is related to the interaction between vestibular and visual systems. In 2D videos this interaction does not take place, whereas in the immersive videos the visual stimulus changes according to the vestibular one.

Shiota and colleagues^4^ postulate a specific psychophysiological pattern of awe, even if it has not been empirically tested yet^24^. Shiota *et al*.^24^ exposed participants to 10 positive emotions including awe, and a neutral control condition. They assessed six psychophysiological variables: Cardiac Interbeat Interval (IBI); Cardiac Pre-Ejection Period (PEP); Skin Conductance Responses (SCRs); Respiration Rate; Respiration Synus Arrhythmia (RSA); Mean Arterial Pressure (MAP). They found that awe led to a lengthening of PEP, compared to all the other conditions, as well as significantly lower levels of SCRs, in contrast with “amusement” and “anticipatory enthusiasm” conditions. Authors argued that PEP patterns of awe were consistent with the “sympathetic withdrawal”, that they had theorized. Importantly, cardiac measures, as well as the SCRs, emerged as one of the most relevant psychophysiological components for differentiating awe from other emotions. Nevertheless, although Shiota *et al*.^24^ and Oveis *et al*.^31^ theorized the role of parasympathetic system in differentiating awe from other emotions, they focused mainly on the sympathetic system.

Here we tested the potential of immersive videos - the highest realistic form of Virtual Reality that excludes the dimensions of navigation^24^ - in inducing awe. In different conditions, we used awe-inducing content and neutral content. The neutral content condition controlled for the effect of awe-inducing content, e.g., ref. 17. We hypothesized that the immersive presentation would increase awe more than a 2-D screen presentation. We also hypothesized that immersive experiences would enhance the feeling of awe more than non-immersive ones. We measured awe using an integrated methodology featuring both self-reported retrospective measures and physiological assessment of awe.

## Results

Analyses were done using IBM SPSS Statistics software (Version 21, release 21.0.0.0 64 bit edition).

### Awe, vastness and need for accommodation

**H1**: *Immersive videos induce more intense awe than 2D screen videos*.

We carried out a repeated measures ANOVA: 2 (media: 2D screen vs. immersive screen) × 2 (content: neutral vs. awe), with self-reported awe as a dependent measure. Results showed a significant main effect of “content” [(F (1,41) = 125.7; p < 0.001, η^2^ = 0.754)]: awe-inducing contents on immersive or on a 2D screen were significantly more awe-inducing than neutral ones displayed on immersive or on 2D screen. More, results indicated a significant main effect of “media” [F(1,40) = 34.793, p < 0.01; η^2^ = 0.153]. Immersive VR displaying awe content or neutral content, compared to the 2D screen videos depicting awe or neutral content elicited a significantly higher sense of self-reported awe. Results showed also a significant interaction effect: there was a more intense sense of awe in awe from immersive video [F(1,41) = 14.133; p < 0.01; η^2^ = 0.256]. In other words, it was the combination of immersion and awe-inducing content that resulted in the highest level of self-reported awe.

**H2:** *Immersive videos induce a significantly higher sense of “perceived vastness” than 2D screen videos*.

We carried out a repeated measures ANOVA: 2 (media: 2D screen vs. immersive screen) × 2 (content: neutral vs. awe) with “perceived vastness” as a measure. Results evidenced significant main effect of “content” [F(1,41) = 91.820; p < 0.01; η^2^ = 0.691]: Awe-inducing stimuli on immersive or on a 2D screen induced a more intense sense of vastness than neutral immersive video and neutral 2D screen video. Finally, there was also a significant main effect of “media” [(F(1,41) = 7.987; p < 0.001; η^2^ = 0.459)]. Results did not show a significant interaction effect [F(1,41) = 1.018; p = 0.339; η^2^ = 0.024]. In other words, immersive videos and awe-inducing video resulted in higher sense of vastness.

**H3:** *Immersive videos induce a significantly higher “need for accommodation” than 2D screen videos*.

We carried out a repeated measures ANOVA: 2 (media: 2D screen vs. immersive screen) × 2 (content: neutral vs. awe), with “perceived need for accommodation” as a measure. Results indicated a significant main effect of “content” [F(1,41) = 12.396; p < 0.001; η^2^ = 0.232]: awe-inducing stimuli displayed both in immersive or on a 2D screen elicited more intense need for accommodation than neutral immersive and 2D video. Moreover, results indicated a significant main effect of “media” [F(1,41) = 18.828 p < 0.001; η^2^ = 0.315]. Finally, we found a significant interaction effect [F(1,41) = 3.85; p = 0.057; η^2^ = 0.086)], that is that immersive VR combined with awe-inspiring content resulted in the most intense awe experience.

### Sense of presence

**H4:** *Immersive videos induce a significantly higher “physical space” and “engagement” than 2D screen videos*.

We carried out a repeated measures ANOVA: 2 (media: 2D screen vs. immersive screen) × 2 (content: neutral vs. awe), with each of the dimensions of presence as measures (i.e., physical space and engagement). There was a main effect of media on physical space [F (1,41) = 150.581; p < 0.001; η^2^ = 0.79]: Awe-inducing immersive and 2D screen videos were able to significantly enhance the perceived sense of being physically present within the virtual environment more than neutral immersive and 2D screen videos.

There was a main effect of “content” on “engagement” [F (1,41) = 53.975; p < 0.001; η^2^ = 0.568]: awe-inducing stimuli displayed both on an immersive or 2D screen elicited more intense “engagement” than neutral immersive and neutral 2D video. Finally, there was also a significant main effect of “media” for “engagement” [F (1,41) = 102.801; η^2^ = 0.715], nonetheless, no significant interaction effect emerged [F(1,41) = 1.907; p = 0.175; η^2^ = 0.44]. These effects were in line with previous findings in literature which demonstrated the ability of VR to manipulate and enhance the general sense of space^46, 47^.

Descriptive statistics on awe, vastness, need for accommodation, sense of presence across conditions are shown in Table 1.

**Table 1:** Descriptive Statistics of Awe, Vastness, Need for Accommodation, Engagement, and Physical Presence.

Conditions	Awe		Vastness		Need for accommodation		Engagement		Physical Presence	
	Mean	SD	Mean	SD	Mean	SD	Mean	SD	Mean	SD
Neutral 2D screen	1.500	0.890	1.815	0.876	1.500	0.800	1.620	0.512	1.853	0.783
Awe 2D screen	3.404	1.530	3.190	1.295	1.793	0.988	2.190	0.718	2.008	0.812
Neutral immersive screen	2.071	1.330	2.107	0.787	2.744	1.570	2.415	0.657	3.018	0.771
Awe immersive screen	5.119	1.533	3.756	1.250	2.738	1.575	3.181	0.636	3.326	0.729

*Note*. n = 42.

### Corroborative measures of awe: psychophysiological measures

We reported psychophysiological results according to the two target dimensions of arousal and valence. Regarding arousal, we reported data concerning sympathetic and parasympathetic activation during video exposure. Results concerning valence referred specifically to the activity of Zygomatic Major Muscle and Corrugator Supercilii Muscle.

Repeated measure ANOVA: 2 (media: 2D screen vs. immersive screen) × 2 (content: neutral vs. awe) was carried out with respect to three main indexes of sympathetic and parasympathetic activation, and two indexes of valence. Two indexes referred to sympathetic activation (i.e., Very Low Frequency measures – VLF; Skin Conductance Responses), and one to the parasympathetic activation (High Frequency measure – HF). One index referred to awe negative valence (EMGa) and one to awe positive valence (EMGb). (Two participants’ physiological recordings were not available due to problems with sensors placement).

#### Sympathetic Autonomous System and awe

We carried out two separated repeated measure ANOVA: 2 (media: 2D screen vs. immersive screen) × 2 (content: neutral vs. awe) for each of the indexes of sympathetic activation. As regard cardiac activity, a significant main effect emerged for sympathetic activation with Very Low Frequency Total Power [F(1,37) = 7.019; p < 0.05; η^2^ = 0.159]: Very Low Frequency Total Power was significantly higher in the awe and neutral immersive condition compared to awe and neutral 2D conditions.

In terms of Skin Conductance Response, there was a significant main effect of “media” [F(1,37) = 4.590; p < 0.05; η^2^ = 0.108]: immersive VR displaying awe content or neutral content, compared to 2D screen videos depicting awe or neutral content induced a significantly greater Skin Conductance.

In other words, immersive VR were able to increase sympathetic activation significantly more than 2D screen videos. Indeed, these findings are in line with researches demonstrating the amplifying role of VR on Skin Conductance^48, 49^. Again, awe content alone did not result as being characterized by a sympathetic activation, thus we chose to deepen this aspect. Therefore, we analyzed the parasympathetic component of awe in order to understand whether it is characterized not only by a sympathetic withdrawal as Shiota *et al*.^4^ found, but also by a cholinergic activation.

#### Parasympathetic Autonomous System and Awe

We carried out a repeated measure ANOVA: 2 (media: 2D screen vs. immersive screen) × 2 (content: neutral vs. awe) with HF (High Frequency) Total Power as a measure. Results indicated a significant effect in the interaction between “media” and “content” [F (1,37) = 5.665; p < 0.05; η^2^ = 0.133]: immersive awe-inducing videos led to a significant increase in Total Power than or neutral immersive video, awe-inducing 2D screen videos or neutral 2D video. Despite this effect, no main effect of medium and content emerged. This confirmed the hypotheses of previous works on the psychophysiology of awe. In short, a sympathetic withdrawal occurred *as well as* a parasympathetic activation. This activation was more intense when awe was induced by immersive videos compared with 2D screen videos.

Descriptive statistics on awe, vastness, need for accommodation, sense of presence across conditions are shown in Table 2.

**Table 2:** Descriptive Statistics of Very Low Frequency Total Power and Skin Conductance Response (Sympathetic indexes) and Total Power (Parasympathetic index).

Conditions	Very Low Frequency Total Power		Skin Conductance Response		Total Power	
	Mean	SD	Mean	SD	Mean	SD
Neutral 2D screen	103.548	236.376	1.745	0.858	366.840	1327.376
Awe 2D screen	90.882	147.919	1.8567	1.066	250.035	775.613
Neutral immersive video	201.194	271.287	2.068	1.146	491.386	1219.688
Awe immersive video	237.309	35.664	2.4428	2.516	905.875	1979.276

*Note*. n = 40.

#### Hedonic Valence of awe

Results showed no main effect for “media” [F(1,38) = 0.323; p = 0.573; η^2^ = 0.08] or “content” [F(1,38) = 3.163; p = 0.083; η^2^ = 0.077] or interaction significant effect occurred between media and content in the Supercilii activity [F(1,38) = 0.493; p = 0.487; η^2^ = 0.013]. Results showed that no main effect for “media” [F(1,38) = 0.767; p = 0.387; η^2^ = 0.020] or “content” [F(1,38) = 1.782; p = 0.179; η^2^ = 0.047] or interaction significant effect occurred between media and content in the Zygomaticus activity [F(1,38) = 0.105; p = 0.748; η^2^ = 0.003]. Taken together, these results could suggest a more complex intrinsic pattern of valence characterizing awe as a unique positive emotion in that awe did not appear to produce facial muscle changes commonly associated with presentation of pleasant stimuli.

## Discussion

“Literature demonstrated that the more immersive the scenario is, the more intense is the subsequent emotional state elicited^9^. Specifically, immersive scenarios can increase the sense of presence, or the illusion of “being there” created by a virtual (or physical) world, thus eliciting strong emotional responses^11, 12^. In other words, presence emerged as an amplifier of emotional responses. Therefore, the primary aim of this study was to test whether a higher realistic form of VR can elicit more intense experiences of awe in the lab. Towards this end, we utilized an integrated methodology combining both retrospective and physiological measures. This combination allowed us to advance the investigation of awe’s physiological correlates. We found that immersive effectively increases the intensity of awe experiences compared to normal 2D videos. Moreover, VR increased the sense of engagement, the sense of physical space, and the perception of vastness, each of which increased self-reported awe.

Furthermore, several physiological measurements were recorded, which resulted in various findings. For Skin Conductance Responses, it was the medium, not the content, that was primarily responsible for alterations on this measure. We also tested Shiota *et al*.’s^4^ hypothesis about a parasympathetic activation of awe and found that awe-inducing content displayed on immersive video elicited a stronger parasympathetic activation compared to other immersive contents. However, this result was in line with Shiota *et al*.^4^ who found a momentary β-adrenergic activation using 2D screen videos. We adopted a methodology able to strengthen awe intensity, and this was evident also from psychophysiological measures. Awe parasympathetic activation emerged more clearly than in Shiota *et al*.^4^. Given the activating potential of immersive VR environments^9, 40^, it is more surprising that the combination of VR and awe-inspiring contents led to a more intense parasympathetic activation, instead of sympathetic one. These findings highlighted the VR was able to elicit a more intense awe even in the lab.

Finally, we did not find any significant difference between Corrugator Supercilii and Zygomatic Major muscles activity. Awe emerged as an “ambivalent” emotion – as far as facial muscle activity goes - in which positive and negative muscle tones were blended. This could be interpreted as the first experimental evidence of the complex nature of valence in awe, as compared with classical models of emotions^50^.

More, VR offers several pathways of exploration for awe researchers. For example, one could alter the dimension of vastness by manipulating the level of presence. Other manipulations are possible, such as altering the level of interactivity in environments. Awe has resulted more as a “stimulus-oriented” emotion (i.e., an emotion induced by non-human elicitors) than a “other-oriented” emotion, (i.e., an emotion elicited by social-interactional stimuli)^17^. However, VR could offer the possibility to investigate the interactional side in a controlled setting. For example, it could be examined how progressively engaging levels of interaction with the environment or with other virtual characters, could enhance the intensity of awe experience or could affect hedonic tune of this experience.

Moreover, VR offers the possibility to investigate the two cognitive appraisals of awe more deeply. For example, as Huron^51^, Silvia^52^ and Chirico *et al*.^5^ indicated, awe can be conceived as a particular form of surprise, specifically regarding the need for accommodation component^5^. The basic mechanism is the violation of expectations, which includes several forms of violations^51, 53^. VR allows creating different versions of expectancy violations. For example, paradoxical scenarios could be reproduced in VR with a high experimental control, such as the illusion of time travelling^54^, or a strong sensorial discrepancy^35^. Further, given the relevance of mental-schema violations, this experimental paradigm could be easily replicated for studying the impact of intense feelings of awe on processes based on this mechanism such as creativity^39, 55^ both at the individual and at the group level^56^.

Furthermore, here we focused mainly on a visual stimulation of awe. However, it could be useful to analyze the impact of other sensorial channels on awe emergence. For example, music has been demonstrated as an effective inductor of awe^51, 53^ and other complex states, e.g., refs 57, 58. However, specific musical features responsible for awe elicitation have not been investigated yet. With this regard, music can be used for two purposes. First, it could be tested how auditory stimuli, such as selected musical pieces, combined with VR could improve awe induction. Second, it could be possible to investigate the best combination between specific musical violations and immersive virtual experiences. Finally, a more visionary perspective could be to visually translate musical features into concurrent visual stimuli creating extremely engaging experiences of awe.

In terms of other measures, VR offers the opportunity for future investigations of the dynamics of awe using neuroimaging. These include Electroencephalography (EEG), Near Infrared Spectroscopy (NIRS), and Functional magnetic resonance imaging (fMRI). In this last case other forms of VR could be used, for example a CAVE in which the participants are physically immersed and surrounded by screens on which the images are back-projected^59^.

Finally, awe has been conceived as one of the key components of a sudden and enduring personal change^60^ which can be supported by the use of VR^61, 62^. Therefore, a future step could be analyzing the long-term effects of VR induced awe. With this regard, it could be insightful to consider a long-term measure of awe such as the variations related to the endocrine system. Specifically, it could be useful detecting enduring changes after awe exposure and not only in terms of awe proneness, as it has been successfully done^22^.

## Limitations

Despite the potential of VR in inducing a more intense version of awe, some limitations exist. This study could be improved mainly regarding a measurement aspect. This research was based on a single-item self-reported measure of awe, in line with literature on this emotion, e.g., refs 4, 15, 21. However, we addressed this issue by integrating the self-reported assessment with a psychophysiological measurement of awe. Nevertheless, we considered only the peripheral system, and did not investigate how this interacts with the central nervous system. More, we focused on two naturalistic awe-inducing contents, but it would be possible to include also social stimuli, such as a crowded space or the presence of a relevant person. Finally, it could be useful also to consider if different naturals scenarios, such as negative natural phenomena, or interactional experiences could impact similarly on awe induction.

## Conclusions

This study has important implications for emotion research methodology. First, VR *does* enhance the intensity of awe experiences in laboratory setting. Second, VR allows researchers to modulate various dimensions of awe induction stimuli, thus teasing apart different subcomponents of awe.

VR offers to opportunity to observe human responses in simulated as well as completely novel environments that feel quite real to participants. We now have the capability to understand how human beings respond to mundane, unusual, dangerous, and awe-inspiring circumstances – all from the safety of the laboratory.

## Materials and Methods

### Participants

The study included 42 participants, who all voluntarily took part in the study (22 females − mean age = 22.82; S.D. = 2.343; 20 males − mean age = 22.3; S.D. = 2.7). Participants were undergraduate students recruited through campus announcements at an Italian University. Participants who (at the time of the experiment) reported vestibular and/or balance disorders were excluded. Only two participants had tried immersive videos using HMDs. Thus, we considered this variable irrelevant for the analysis. The experimental protocol was approved by the Ethical Committee of the Università Cattolica del Sacro Cuore prior to data collection. Each participant provided written informed consent for study participation. Written consent and all methods were carried out in accordance with the Helsinki Declaration.

### Stimuli

#### Stimuli Selection

Awe-inducing and neutral content was used. This content was selected after a preliminary study that tested the effectiveness of various content for awe elicitation in a separate sample of 36 participants (Chirico *et al*., in press)^63^. In this study, participants watched at 4 video contents: (i) amusing; (ii) awe-inspiring 1 (showing a grand vista on the mountains); (iii) awe-inspiring 2 (depicting a scene of tall trees in a forest); (iv) neutral (hens wandering on grass). Each participant watched at each video once in a counterbalanced order. Participants then rated the extent to which they experienced several different emotional states such as Anger, Awe, Amusement, Disgust, Fear, Pride, Sadness, and Joy. Each of these videos was created using ShotCut video-editing free online tool. Results indicated that the video depicting a scene of tall trees in a forest was the most effective for eliciting awe, and that video of hens wandering on grass did not induce awe (i.e., this video elicited low levels of each assessed emotion) - thus we used these two videos for the current study.

### Selected stimuli and contents

The two videos chosen – an awe-inducing video and a neutral video - were manipulated to the two different mediums of display. Each video was displayed as immersive VR or on a 2D screen. This resulted in 4 conditions:

- Neutral video on a 2D screen;
- Awe-inducing video on a 2D screen;
- Neutral video on immersive screen;
- Awe-inducing video on immersive screen.

Specifically, each of the four videos was composed of the following subsections: (i) a black screen lasting 6000 milliseconds; (ii) a sound (lasting 500 milliseconds) that served as a signal to the experimenter to start physiological recordings; (iii) a black screen lasting 8000 milliseconds after the sound; (iv) the beginning of the video.

Videos were displayed using Samsung Gear VR, a head mounted virtual reality display. Each video lasted 2 minutes (excluding the i, ii, iii subsections). KolorEyes App was used to manipulate the dimension of immersion, by using the “immersive” option (to activate immersive display) or “2D” to display video on a 2D screen. Kolor Eyes 1.5 App is a free immersive video-player for Windows, Mac, HTML5, iOS and Android. This app allows tracking participants’ head orientation both in the 2D screen condition and in the immersive video condition.

### Measures

#### Self-reported measures

After video exposure, participants were required to report the extent to which they experienced awe, presence, the sense of vastness and the need for accommodation, as follows:

(i) Awe was assessed with a single item likert self-report measure among other items measuring eight distinct emotions (from 1 = not at all; to 7 = extremely): Anger; Awe; Disgust; Fear; Pride; Sadness, Amusement and Joy. This questionnaire was used to obtain a measure of “*global perceived awe*”.
(ii) Presence was assessed using two sub-scales (“*Engagement*” and “*Physical Space*”) of the ITC-Sense of Presence Inventory (ITC-SOPI)^43^. The ITC-SOPI is a 42-items on a 5-point Likert scale (1 = strongly disagree; 5 = Strongly agree) questionnaire. This questionnaire is composed of four subscales which demonstrated good internal consistency, showing a Cronbach Alpha ranging between 0.76 and 0.94: Sense of Physical Space (0.94); Engagement (0.89); Ecological Validity (0.76); Negative Effects (0.77). We focused on the two subscales of Physical Space and Engagement since they have already resulted relevant regarding emotional intensity.
(iii) *Perceived vastness* was assessed using four items: 1. What I watched provided me with a deep sense of vastness; 2. I felt small in front of what I watched; 3. I felt meaningless in front of what I saw; 4. I felt my sense of self diminish in front of what I saw). Cronbach Alpha = 0.77.
(iv) *Perceived* need for accommodation was assessed using four items: 1. It was hard to grasp what was going on in the video; 2. I felt confused and bewildered in front of what saw; 3. I was struck by the video). Cronbach Alpha = 0.81.

This questionnaire, which included perceived vastness and perceived need for accommodation dimensions, was created according to the guidelines provided by Schurtz *et al*.^23^ and Piff *et al*.^15^.

#### Psychophysiological measures

The ongoing experience of awe was assessed through physiological measures that have been used in previous studies on awe^24, 31^. Since our approach is new, we sought to corroborate the assessment of awe with self-report and physiological measures. Our aim was two-fold. First, we chose to draw from previous findings for detecting awe using physiological measures. Moreover, we decided to advance the psychophysiological knowledge on awe by paying more attention to the role of the parasympathetic system. To these ends, we measured peripheral nervous system (PNS) activation by using various wearable noninvasive biosensors:

- A biosensor to record Skin Conductance Response (SCR). SCR depends on the activity of the sweat gland which is controlled by the sympathetic nervous system. It is an index of psychophysiological arousal^64^. We recorded SCRs with two electrodes placed on the palmar surfaces of the distal phalanges of the index and ring fingers of the dominant hand. Skin Conductance (SC) is expressed in microsiemens (µS) representing the average of the cleaned signal during a given experimental epoch.
- Blood Volume Pulse (BVP) is a signal obtained through a photoplethysmograph biosensor, which measures fluctuations in blood volume in a specific tissue with a light-emitting diode. The amount of infrared light transmitted to the photoplethysmograph is a function of the amount of blood saturating specific tissue regions. BVP was recorded to measure complex cardiovascular activity to gather information on sympathetic and parasympathetic activations during experimental epochs.
- Two surface electromyography (sEMG) biosensors recorded muscular automatic micro-contractions of both the Corrugator Supercilii Muscle (following corrugator) and the Zygomatic Major Muscle (following zygomatic). Corrugator activity is sensitive to unpleasant stimuli^65^, and does not depend on the awareness of the eliciting stimulus^66^. We used this measure as an index of positive and negative emotional valence as Zygomatic activity has been shown to respond to pleasant stimuli^65, 67^.

A ProComp Infinity 8-channel (Thought Technology Ltd, Montreal, Canada) was used to record all physiological measures during each video session (the experimental epochs), with a sampling rate at 256 Hz for BVP and SCR and at a 2048 Hz for the two EMG. Heart rate variability (HRV) measures were calculated through a custom script in Matlab 7.10.0 (R2010a).

Inter-Beat Interval (IBI) was extracted from the Blood Volume Pulse sensor. It consisted in a measure comparable with the R-R peaks interval extracted from the electrocardiogram. According to the guidelines of Task Force of the European Society of Cardiology and the North American Society of Pacing and Electrophysiology^68^, typical temporal and spectral HRV measures (by the means of Fourier spectral methods) were extracted to evaluate the response of the autonomic nervous system. The rhythms were considered as very low frequency (VLF < 0.04 Hz), low-frequency (LF, 0.04 to 0.15 Hz), and high frequency (HF, 0.15 to 0.4 Hz) oscillations.

About the sEMG, since the raw electromyography is a collection of positive and negative electrical signals, their frequency and amplitude provide information on the contraction or rest state of the muscle. Amplitude is measured in microvolts (μV). As the subject contracts a muscle, the number and amplitude of the lines increase, and, as the muscle relaxes, the number and amplitude of the lines decrease. We considered the Root Mean Square (RMS) to rectify the raw signal and converted it to an amplitude envelope. According to Blumenthal and colleagues^69^, facial EMG corrugator and zygomatic can be considered the best measure for negative and positive emotion valence, respectively.

### Procedure

**Figure 1:**
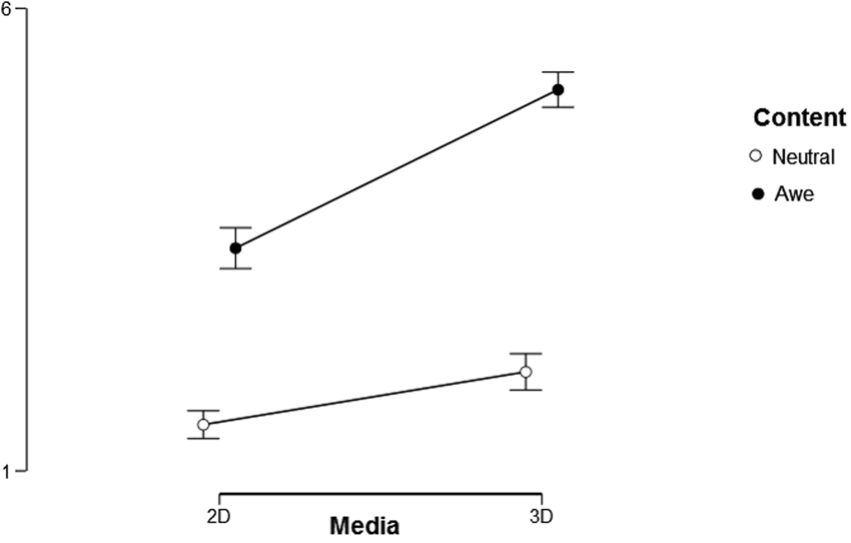
Interaction effect of media and content with awe as a measure. Error bars indicate standard errors of the means.

**Figure 2:**
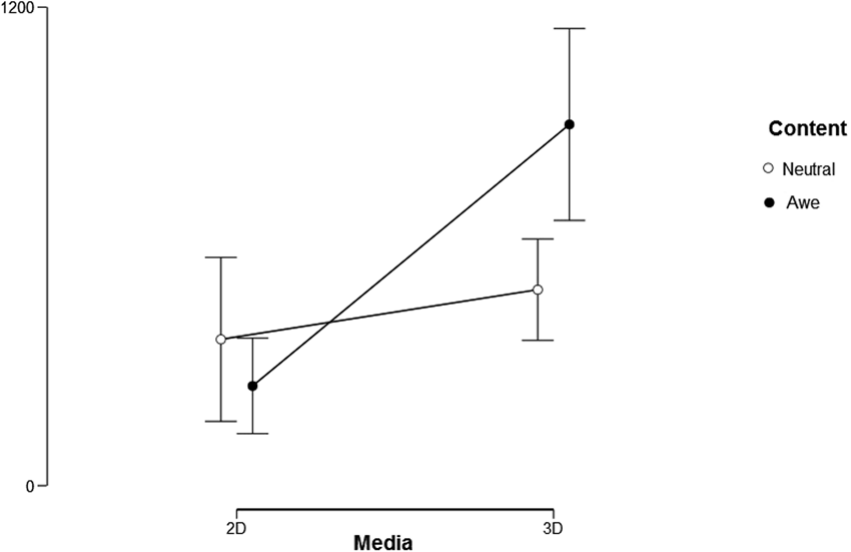
Interaction effect of medium and condition with HF Total power as a measure. Error bars indicate standard errors of the means.

**Figure 3:**
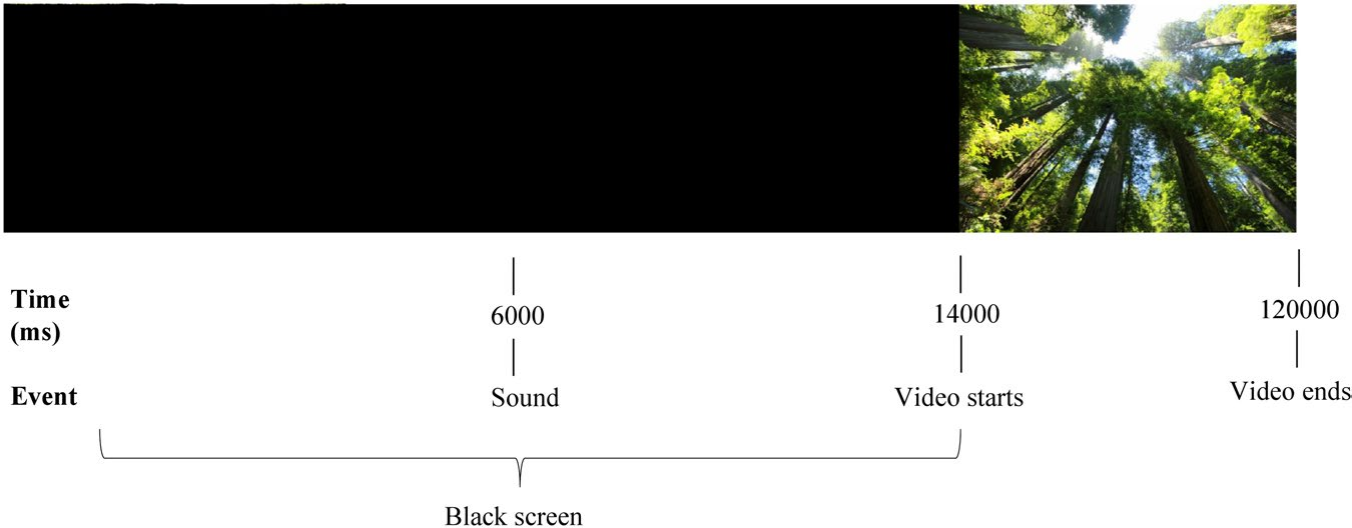
The sequence of video subsections. Image of Tall trees was taken from Pixabay (credits: Pixabay, https://pixabay.com/it/sequoia-foresta-redwood-274158/).

First, participants provided informed consent document. Then, participants were provided both by a written and an oral description of the study, and sensors for physiological measurement were applied. The protocol included 4 video-viewing trials. Each participant watched each video once in a counterbalanced order. During video exposure, cardiovascular activity (with BVP), electrodermal response (with SCR), and facial muscular activity (corrugator and zygomatic) were recorded. Specifically, a baseline measure was obtained (3 min length) while they were sitting comfortably. Participants then put on a virtual reality head-mounted display (i.e., Samsung Gear VR for Samsung Galaxy Note 4) and they received standardized instructions about how to use VR. When participants indicated that they were ready to begin Figs 1, 2 and 3 the experimenter touched the lateral pad to start the video. After each video exposure, participants completed the self-report ratings described above. This procedure was repeated four times, one time for each condition, with each participant. Participants were instructed to explore the video freely and to have their arms lie in the same position for all the experimental session. The entire experiment lasted about 55 minutes.
